# Pirate Stealth or Inattentional Blindness? The Effects of Target Relevance and Sustained Attention on Security Monitoring for Experienced and Naïve Operators

**DOI:** 10.1371/journal.pone.0086157

**Published:** 2014-01-21

**Authors:** Erika Näsholm, Sarah Rohlfing, James D. Sauer

**Affiliations:** University of Portsmouth, Portsmouth, Hampshire, United Kingdom; University of Akron, United States of America

## Abstract

Closed Circuit Television (CCTV) operators are responsible for maintaining security in various applied settings. However, research has largely ignored human factors that may contribute to CCTV operator error. One important source of error is *inattentional blindness* – the failure to detect unexpected but clearly visible stimuli when attending to a scene. We compared inattentional blindness rates for experienced (84 infantry personnel) and naïve (87 civilians) operators in a CCTV monitoring task. The task-relevance of the unexpected stimulus and the length of the monitoring period were manipulated between participants. Inattentional blindness rates were measured using typical post-event questionnaires, and participants' real-time descriptions of the monitored event. Based on the post-event measure, 66% of the participants failed to detect salient, ongoing stimuli appearing in the spatial field of their attentional focus. The unexpected task-*irrelevant* stimulus was significantly more likely to go undetected (79%) than the unexpected task-*relevant* stimulus (55%). Prior task experience did not inoculate operators against inattentional blindness effects. Participants' real-time descriptions revealed similar patterns, ruling out inattentional amnesia accounts.

## Introduction

Reliance on Closed Circuit Television (CCTV) surveillance is increasing [1]–[3]. Surveillance operators are responsible for maintaining the security of critical infrastructure (e.g., airports and government buildings) and public spaces (e.g., streets and shopping malls). Research on CCTV has typically evaluated technological effectiveness (e.g., image quality; see [2]), operators' ability to match CCTV images of culprits with live or photographed suspects (e.g., [4]), or the impact of CCTV prevalence on crime rates and public perceptions of security (e.g., [5]). While the importance of human factors in CCTV operating has been identified [6], [7], research investigating human performance in the CCTV monitoring context is scarce [2], [8]. This is surprising as ineffective monitoring can have serious consequences. The failure to detect criminal targets or events and intervene appropriately not only facilitates criminal activity but also fosters negative public perceptions of and apathy towards security measures. We investigated the effects of three factors (i.e., inattentional blindness, sustained attention and prior task experience) on CCTV monitoring performance.

While CCTV systems are often used for post-hoc analyses of events [9], real-time monitoring is essential for public protection, and operational responses must be initiated when illegal activity is detected. According to Scott-Brown and Cronin [9], the key to successful real-time monitoring is to maximize detection for unexpected events. However, research demonstrates the limits of humans' capacity to detect and identify unanticipated target stimuli. For example, Wolfe, Horowitz, and Kenner [10] examined error rates in artificial simulated luggage-screening task. At the prevalence of 50% (i.e., targets were presented in 50% of trials) the ‘miss’ rate was 7%. However, when prevalence rates dropped to 10% and 1% of trials, miss rates rose to 16% and 30%, respectively (see also [11], [12]). These error rates raise questions regarding the effectiveness of participants' real-time monitoring ability, and highlight difficulties associated with the detection of unexpected events. Here, we discuss how one particular attentional phenomenon – inattentional blindness – may contribute to detection failures.

Inattentional blindness refers to a failure to detect unexpected stimuli, even when these stimuli are conspicuous. When attention is directed toward a primary task, observers may fail to perceive otherwise salient visual features of their environment [13]. Inattentional blindness has been demonstrated both in basic perception tasks (e.g., [13]) and for complex, dynamic stimuli (e.g., [14], [15]).

In their classic demonstration of inattentional blindness, Simons and Chabris [15] showed participants footage of two teams of three individuals moving and passing a basketball. Participants were instructed to count the number of passes made by one of the teams. After approximately 50 seconds, either a woman with an umbrella or a person wearing a gorilla suit walked through the game. Of the 192 participants, 46% failed to detect these unexpected events (56% failed to detect the gorilla, 35% failed to detect the woman). More recently, Chabris, Weinberger, Fontaine and Simons [16] demonstrated inattentional blindness in real-world conditions. Participants ran after a confederate, counting the number of times the confederate touched his head. At night, 65% of participants failed to notice a staged fight taking place along the running route. During the day, 44% of a second set of participants failed to notice the fight. These findings demonstrate that individuals engaged in a primary task often fail to notice otherwise salient stimuli in their environment if these stimuli are not relevant to the primary task.

These findings demonstrate the importance of inattentional blindness for CCTV monitoring, where the ability to detect and monitor events is essential. However, to our knowledge, no research has *directly* investigated the topic. Stedmon, Harris, and Wilson [17] found that a high percentage of participants monitoring CCTV footage failed to detect a significant event, and discussed their finding with a brief, post-hoc reference to inattentional blindness (although the authors actually referred to change blindness, the phenomenon under discussion would be more appropriately described as inattentional blindness [18]). However, while Stedmon et al.'s findings may indicate inattentional blindness in a CCTV monitoring context, their research was not a direct exploration of the issue, and boundary conditions for inattentional blindness require further examination.

We examined inattentional blindness in real-time CCTV monitoring, and explored three potential boundary conditions for inattentional blindness in this setting, selected on the bases of their theoretical and applied value. Specifically, we investigated the effects of (a) the relevance of the unexpected event to the primary task, (b) length of sustained attention, and (c) operators' prior task experience on inattentional blindness rates. Research suggests that unattended stimuli that share features with task-relevant stimuli are less likely to produce inattentional blindness than those that do not [19]. Models of visual selective attention demonstrate that visual stimuli must compete for attentional resources (e.g., [20]–[22]). Folk, Remington, and colleagues [23], [24] found that, in basic perception tasks, attentional control settings and attention capture are largely contingent on task demands. Further, Braun and Julesz [25] argued that observers are able to detect and discriminate items outside their attentional focus, but that stimulus saliency (influenced by observer expectation) determines the level of visual processing that a stimulus receives (see [26]). Similarly, Most, Scholl, Clifford and Simons [27] reported that the most influential factor affecting detection of unexpected objects is the individual's attentional set (i.e., the individual's readiness to receive specific type of information). If task goals determine individuals' attentional sets and influence their readiness to perceive stimuli, unexpected but task-*relevant* stimuli would be predicted to elicit lower levels of inattentional blindness (i.e., higher detection rates) than unexpected task-*irrelevant* stimuli. We explored this by manipulating the task-relevance of our unexpected stimuli. Our primary task required participants to monitor simulated CCTV footage for ‘suspicious’ activity, and verbalize any suspicious activity detected (as if reporting their observations to a colleague approaching the scene). The unexpected stimulus was either an individual entering the scene and placing a package on the ground before exiting the scene (task-*relevant*), or a pirate (of the eye-patch and parrot variety) entering and exiting the scene (task-*irrelevant*). Given the prevalence of warnings relating to unattended baggage and parcels in public places (and the relative scarcity of pirates in public places), we expected the package-related stimulus to be of greater relevance to the primary, security-oriented task.

CCTV operators are often required to sustain attention over extended time periods [28]. Thus, sustained vigilance is fundamental to effective monitoring performance. However, research has repeatedly shown that detection rates decrease over time in applied monitoring settings. For example, train drivers become less likely to detect vital railway signals [29], and CCTV operators are more likely to miss vital visual information [8]. Parasuraman et al. [30] reported that detection of threat-related targets (i.e., an individual reaching for/using a gun vs. a hairdryer) also declines over time under visually degraded conditions (but not under non-degraded conditions). While these performance declines are thought to reflect increased cognitive workload, Surette [31] argued that real-time monitoring might lead to intense feelings of boredom. Consequently, critical events might be missed as a result of inattention. Previous inattentional blindness research has exclusively involved sequences that take place over relatively short time periods. For example, the clips used in Simons and Chabris' [15] study were 75 s long. To advance this area of research and increase its applied relevance, we investigated the effects of sustained monitoring on inattentional blindness by manipulating the length of footage participants were required to monitor. Participants monitored footage for either 2 or 43 minutes (approximately).

The vulnerability of experienced (cf. naïve) operators to inattentional blindness has not been extensively investigated. However, Memmert [32] showed Simons and Chabris' [15] gorilla video to participants who had either played basketball for more than ten years or who were novices to the game. Experienced basketballers were significantly more likely to detect the gorilla, compared to novices. Further, when an individual practices the primary task (e.g., tracking basketball passes) prior to a critical trial (i.e., a trial including the unexpected event), inattentional blindness rates decline (see [33], [34]). Similar to expertise, prior task experience is thought to reduce the attentional demands of the primary task (i.e., cognitive load), increasing the attentional resources available for detecting and processing the unexpected event [27], [35], [36]. Our operationalization of operator experience differed from those reported above. Previous operationalizations reflect experience in the activity being monitored (e.g., playing basketball), or training with the specific stimuli being monitored. We operationalized experience in terms of prior experience monitoring CCTV footage. Recent research by Drew, Vo, and Wolfe [37] suggests that prior monitoring-task experience may, at least partially, attenuate inattentional blindness effects. Drew et al. had experienced radiologists and naïve operators monitor a series of chest computed tomography (CT) slides for the presence of lung nodules. During the series, an unexpected stimulus (an image of Simons & Chabris' gorilla) faded into and out of visibility. While scanning for lung nodules, 83% of experienced operators and all of the naïve operators failed to report detecting the unexpected stimulus, demonstrating clear inattentional blindness despite detection rates near ceiling (88%) under control conditions. However, while both experienced and naïve operators showed evidence of inattentional blindness, Drew et al.'s findings suggest that prior task experience may offer some (albeit limited) protection against detection failures. Basic detection research also demonstrates that prior experience with a task paradigm can protect against declines in detection performance associated with the attentional demands of executing a concurrent monitoring task (e.g., [38]). Braun found that participants with “extensive prior experience with tachistoscopic displays” (p.424) but no training specific to that particular experiment outperformed novice participants in a ‘Popout’ detection task. Further, detection performance for experienced participants was comparable to performance for participants who received substantial training specifically related to that experiment (trained participants completed thousands of practice trials prior to data collection). Braun argued that extensive prior experience with a task may facilitate a “relatively direct route from preattentive processing to perceptual report”, improving experienced observers' performance (cf. novice observers) under conditions of increased attentional load (p.425; see also [39], for perceptual encoding advantages related to expertise). Prior experience with the general task paradigm did not protect against the effects of increased attentional load in all conditions, but it produced results similar to task-specific training. Given that task-specific training has been shown to reduce inattentional blindness, prior experience with the task may offer similar benefits. Braun demonstrated that manipulations designed to impair basic processes underlying target detection can differentially affect experienced and novice participants. However, the generalizability of these basic effects to more applied monitoring tasks has not been empirically assessed. Thus, we compared levels of inattentional blindness in a group of CCTV novices with a group of experienced CCTV operators (infantry personnel who monitor CCTV footage in a professional security setting). Prior monitoring-task experience may improve detection rates by facilitating more efficient monitoring/search strategies, reducing task difficulty and associated cognitive processing demands, or through effects on observer expectations [37]–[39].

Our methodology emulated Simons and Chabris' [15] with two important modifications. First, our stimulus clip included simulated criminal activity designed to be relevant to a CCTV monitoring setting. Second, our primary task required participants to verbalize any observed aggressive and suspicious behavior. Inattentional blindness is typically assessed by establishing a primary task and then asking participants, after viewing the stimulus clip, if they saw the unexpected target while completing the primary task (e.g., Simons and Chabris asked participants a series of cued recall questions including “Did you see a gorilla?”). Unavoidably, this method of measuring inattentional blindness assesses participants' *memory* for seeing the unexpected stimulus. This is distinct from assessing whether or not the participant detected the unexpected stimulus at the time it was presented, and memory errors may inflate inattentional blindness rates (cf. [15]). In addition to using a post-event recall measure, we analyzed participants' verbalizations to determine whether or not the unexpected stimulus was reported during real-time monitoring. This innovative approach provided a measurement of inattentional blindness free of any memory-related effects (cf. [40]).

Based on previous research demonstrating inattentional blindness, we expected to find inattentional blindness in the current study (*Hypothesis 1*). Previous research has implicated attentional set and task demands in inattentional blindness. Thus, we hypothesized that an unexpected task-relevant stimulus would be detected more often than an unexpected task-irrelevant stimulus (*Hypothesis 2*). Further, given that detection rates typically decrease as the length of the monitoring period increases, we expected levels of inattentional blindness to be higher for participants viewing the longer version of the footage, compared to those viewing the shorter version (*Hypothesis 3*). Finally, we compared the performance of experienced and naïve CCTV operators. Stimulus-specific training has been shown to reduce inattentional blindness, and detection research has demonstrated that task familiarity and stimulus-specific training offer similar benefits (cf. non-trained, naïve participants) for detection under conditions of increased attentional load. However, it is unclear if these findings will generalize to the present context.

## Methods

### Ethics statement

The Psychology Department Research Ethics Committee, University of Portsmouth, UK, reviewed and approved this experimental protocol. Participants provided full written consent prior to participating and were fully debriefed upon completion.

### Participants and Design

We used a 2(naïve vs. experienced operator) ×2(CCTV clip length: 2 vs. 43 minutes) ×2(stimulus relevance: relevant vs. irrelevant) between-subjects design. The experienced operators were 88 infantry personnel stationed overseas on active duty, who regularly monitor CCTV footage as part of their duties. This group was comprised of males aged 18 to 37 years (*M* = 23, *SD* = 4). CCTV monitoring experience ranged between 1 and 19 years (*M* = 5, *SD* = 4). On average, infantry personnel may be expected to monitor CCTV footage for four to eight hours per month. Using the conservative estimate, this equates to an approximate range of 48 to 912 hours of monitoring experience (M = 250, SD = 204). Experienced operators participated on a voluntary basis. 88 students and staff (67 female) were recruited from a UK university as the novice group. Ages ranged from 18 to 57 years (*M* = 24, *SD* = 10). Participants in the novice group had no experience monitoring CCTV footage. Students earned course credit and staff participated on a voluntary basis. Five participants failed to follow instructions (did not comply with the primary, verbalization task) and were excluded from analyses. The final sample consisted of 171 participants (84 experienced and 87 naïve operators). Within each level of operator experience, participants were randomly allocated to clip length and stimulus relevance conditions.

### Materials

Two stimulus clips were produced for this study. Four females and one male, aged between 19 and 24, were recruited as actors. Each clip involved a primary event and an unexpected (task-*relevant* or -*irrelevant*) stimulus. The primary event lasted 50 seconds and was filmed in daylight in an alleyway. To permit comparison with Simons and Chabris' [15] stimuli, our clips included a dynamic primary event involving a group of people interacting. Simons and Chabris [15] reported that observers were less likely to detect the gorilla (a dark-colored stimulus), when the attended basketball team was dressed in white (i.e., when basic visual features differed between the attended event and the unexpected stimulus). In our event, actors in the primary event were dressed in dark clothing and the unexpected target was dressed in light clothing.

The primary event featured a female (A) arriving at the location with a bike. Subsequently, two other females (B and C) and a male (D) arrived. A acted aggressively (e.g., pushing) towards B, C, and D (and vice versa). Subsequently, A interacted with C and D to execute an apparent drug deal, while B stole A's bike. After approximately 30 seconds of interaction, the unexpected stimulus entered the shot. In the *relevant* condition, a female walked halfway across the back of the scene, placed a suspicious parcel on the ground, stood up, looked straight into the camera, and walked off. This stimulus was deemed relevant to the task as CCTV operators will often look for suspicious packages left in public places. In the *irrelevant* condition, the same female appeared in the back of the scene wearing a pirate's costume (light clothing, a pirate hat, eye patch and a parrot on her shoulder). The pirate entered scene, looked straight into the camera and exited. In both versions, the unexpected stimulus was visible for 9 seconds and, after the target had exited, the four other individuals ran off. Actors in the primary event did not engage with, or appear to notice, the target. Rehearsal prior to recording ensured parity between the two clips. Pilot testing confirmed that participants were able to detect the unexpected target (e.g., pirate or woman with a parcel) under conditions of full attention. 20 participants (10 each for task-*relevant* and -*irrelevant* stimuli) viewed the short version of the event, and were asked to attempt to detect the pirate/woman with a parcel. Detection rates were perfect.

Clip length was manipulated by adding footage prior to the primary event, and involved three stages. First, we filmed the critical event (including the appearance of the unexpected target). Second, we filmed an additional 42 minutes of footage of the area where the critical event took place. Third, we added this additional footage to the beginning of the critical event. For the long clip condition, we simply combined these two lots of footage (i.e., added 42 minutes footage at the beginning of the critical event). For the short clip condition we added only the final one minute of the 42 minutes additional footage to the beginning of the critical event. The additional footage showed non-target pedestrians walking through the alleyway at sporadic intervals. Although the monotony of this footage may have encouraged lapses in attention, it provided a realistic approximation of common conditions under which operators are required to sustain vigilance [31]. For the short clips, the additional footage contained no non-target individuals. The additional footage for the long clips contained 17 non-target individuals. Short clips were 1 minute and 50 seconds in duration and long clips lasted for 42 minutes and 50 seconds. In all conditions, the unexpected target entered shot 20 seconds before the footage ended. All clips were muted. These stimulus materials are available online at https://openscienceframework.org.

### Procedure

Participants were tested individually either on university premises (novices), or at the army base (experienced operators). Prior to viewing the clip, participants were instructed to imagine being a CCTV operator responsible for the security of an outdoor location. Anything that could potentially be a threat to security, for example aggressive or suspicious behavior, was to be described verbally (into a Dictaphone), in real-time, to a (hypothetical) colleague ‘on the ground’. Pilot testing confirmed that participants could understand and follow the instructions.

Clips were presented in color, in full screen on 15 or 20 inch monitors. Screen size varied within conditions, and did not affect detection rates. After viewing the footage participants completed a short questionnaire. In an effort to ensure that participants who detected the unexpected stimulus would report it, questions were structured to probe for increasingly detailed responses. Further, to counter demand effects inherent in the leading nature of the questions asked, and to verify the accuracy of any ‘yes’ responses (i.e., indicating that the unexpected stimulus had been detected), participants were required to provide details of what they had seen. Participants in the relevant conditions were also asked questions regarding what, if anything was left on the scene (i.e., the parcel).

## Results

### Data Preparation

Audio files were transcribed verbatim. Transcript content was used to verify task engagement. Five participants failed to verbalize anything relating to the critical event, and their data was discarded. The remaining 171 participants all verbalized at least one sentence relating to the critical event. Transcripts also allowed an alternative method of testing for inattentional blindness (cf. the post-event recall measure).

Participants' answers were coded for (i) whether the unexpected stimulus was detected/undetected, and (ii) correct/incorrect description of stimulus. Two raters initially coded 34 (approximately 20%) of the questionnaires. Discrepancies were identified and resolved via discussion. Following agreement on the initial 34 questionnaires the two raters split the remaining 137 questionnaires. The dataset for this experiment is available online at https://openscienceframework.org.

Gender ratios clearly differed between the experienced and naïve operator groups. However, crosstabulation analyses within the naïve group data found no significant gender differences on any measure of detection, *χ* ^2^ (1, *N* = 87) <0.859, *p*>.431, strongly suggesting that gender differences between groups did not contribute to the results obtained.

### Post Event Measures of Inattentional Blindness

Of the 171 participants, 66% (*SE* = 4) did not report detecting the unexpected stimulus. This finding demonstrates inattentional blindness in the context of CCTV monitoring, supporting *Hypothesis 1*. Table 1 displays the percentages of participants failing to detect the unexpected stimulus in each condition.

**Table 1:** Percentage (SE) of Experienced and Naïve Operators Failing to Detect the Unexpected Stimulus According to Clip Length and Event Relevance, Based on the Post-Event Questionnaire (Strict Coding Scheme).

		Stimulus Relevance					
		Relevant			Irrelevant		
Operator	Clip Length	%	(*SE*)	*n* a	%	(*SE*)	*n* a
Experienced	Long	64	(10)	22	80	(9)	20
	Short	57	(11)	21	71	(10)	21
	Overall	61	(7)	43	76	(7)	41
Naïve	Long	41	(10)	22	91	(6)	21
	Short	59	(10)	22	73	(10)	22
	Overall	50	(8)	44	81	(6)	43
Overall	Long	52	(8)	44	85	(6)	41
	Short	58	(8)	43	72	(7)	43
	Overall	55	(5)	87	79	(5)	84

^a^ This value refers to the total number of participants in the cell, not to the number of participants who failed to detect the target.

A 2(naive vs. experienced operators) ×2(clip length) ×2(relevance) hierarchical loglinear analysis [41] on detection rates (based on the recall measure) identified a significant association between stimulus relevance and inattentional blindness. Supporting *Hypothesis 2,* participants were less likely to detect the *task-irrelevant* unexpected stimulus compared to the *task-relevant* unexpected stimulus, *χ* ^2^ (1, *N = 171*)  = 10.53, *p*<.001, *w* = 0.25. Based on Cohen's criteria, this represented a small-moderate effect. The non-significant Relevance × Clip length × Operator Experience interaction, *χ* ^2^ (1, *N = 171*)  = 1.60, *p* = .207, indicated that the *task-relevant* stimulus was detected more often than the *task-irrelevant* stimulus across conditions. Clip length was not significantly associated with inattentional blindness, *χ* ^2^ (1, *N = 171*)  = 0.19, *p* = .665, w = 0.03, and there was no significant difference between experienced and naïve operators, *χ* ^2^ (1, *N = 171*)  = 0.11, *p* = .746, w = 0.02. Given the large range in operator experience, two sets of crosstabulation analyses compared detection performance within the experienced operator group. Median split analyses revealed no systematic or significant differences in detection rates between subsets. Similarly, when analyses compared operators with greater (N = 16) or less (N = 68) than 10 years experience, any differences were small and non-significant (*p*>.118, *w*<0.18).

Closer inspection of the data revealed an interesting trend. Some participants reported detecting the unexpected stimulus, but failed to correctly identify it (e.g., describing it in one case as a “*man with a saxophone”*). This suggested that participants may have detected the unexpected stimuli, but that the strict criterion for detection being applied – requiring that participants be able to detect *and* accurately identify/describe the stimuli – may have inflated inattentional blindness rates (this “detection without identification” phenomenon is addressed in more detail below). A more lenient coding scheme – where any reference to a character in the background was coded as a positive detection of the stimulus – increased detection rates. However, inattentional blindness was still evident: 39% (*SE* = 4) of participants failed to detect the unexpected stimulus. A 2(operator experience) × 2(clip length) × 2(relevance) hierarchical loglinear analysis again revealed a significant, moderate-strength association between relevance and detection rates, *χ* ^2^ (1, *N = 171*)  = 15.62, *p*<.001, *w* = 0.50. As above, the *task-irrelevant* unexpected stimulus was significantly more likely to go undetected (54%, *SE* = 5) than the *task-relevant* unexpected stimulus (24%, *SE* = 5). No other associations were significant, *χ* ^2^ (1, *N = 171*) <3.67, *p*>.055.

### Analyses of Inattentional Blindness Based on Transcripts

**Figure 1:**
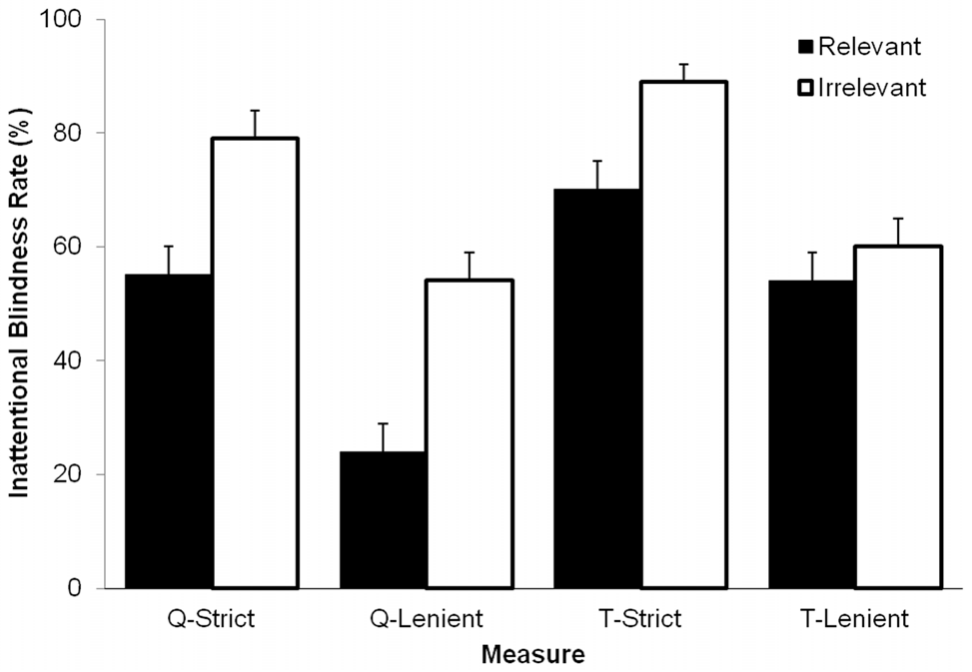
Inattentional blindness rates according to event relevance. Percentage of participants failing to detect the unexpected stimulus according to event relevance based on questionnaire (Q) and transcript (T) data, using both strict and lenient coding schemes. Error bars represent standard errors.

Participants may have detected the unexpected stimulus during the event but, for various reasons, failed to report it on the post-event questionnaire. To address this issue we coded transcribed verbalizations for mention of the unexpected stimulus. Again, we used both strict and lenient criteria for detection. Analyses based on the strict coding scheme produced results consistent with those reported for the post-event measures. Overall, 80% (*SE* = 3) participants failed to verbalize detection of the unexpected event, and the *task-irrelevant* event was more likely to go undetected (89%, *SE*  = 3) than the *task-relevant* event (70%, *SE*  = 5), *χ* ^2^ (1, 171)  = 9.65, *p* = .002, *w* = 0.24. All other associations were non-significant, *χ* ^2^ (1, 171) <1.74, *p*>.187. Analyses based on the lenient coding scheme again found inattentional blindness – 57% (*SE* = 4) of participants failed to detect the target – but found no significant difference between inattentional blindness rates for the *task-irrelevant* (60%, *SE*  = 5) and *task-relevant* events (54%, *SE*  = 5), *χ* ^2^ (1, 171)  = 0.782, *p* = .376, *w* = 0.07. All other associations were also non-significant, *χ* ^2^ (1, 171) <2.29, *p*>.130. Figure 1 presents a summary of relevance effects across analyses.

### Detection without Identification

A number of participants detected the unexpected stimulus but were unable to correctly identify it. We calculated detection without identification rates (i.e., cases for which the strict criterion indicated inattentional blindness but the lenient criterion indicated detection) based on both verbalization and post-event recall data. McNemar's test (a repeated samples analysis for non-parametric data) found no significant differences between overall detection without identification rates (i.e., as a proportion of the total number of trials) based on verbalizations (22%, *SE*  = 3) compared to post-event recall questionnaires (28%, *SE*  = 3), *McNemar's χ* ^2^ (1, 171)  = 2.27, *p* = .132. However, detection without identification accounted for a significantly greater proportion of detection failures on the post-event recall questionnaires (41%, *SE*  = 5), compared to the verbalization data (26%, *SE*  = 4), *McNemar's χ* ^2^ (1, 110)  = 9.32, *p* = .003.

## Discussion

We demonstrated inattentional blindness for naïve and experienced CCTV operators using both real-time verbalization measures and post-event recall measures of detection. Further, the effect persisted even when more lenient criteria for detection – representing a more conservative test of inattentional blindness – were applied. Inattentional blindness rates were lower (detection rates were higher) when the unexpected stimulus was relevant to the primary monitoring task than when the unexpected stimulus was irrelevant to the primary monitoring task. However, contrary to expectations, inattentional blindness rates did not increase when the task required longer periods of monitoring. Importantly, both experienced and naïve operators demonstrated inattentional blindness. Detection rates based on the post-event questionnaire were higher than those based on the transcripts, supporting previous work (e.g., [15], [42]) suggesting that inattentional blindness cannot be attributed to memory failure (cf. inattentional amnesia, [40]). These findings extend previous research (e.g., [15]) to demonstrate that inattentional blindness is a robust phenomenon in applied monitoring contexts, and that observer expectations influence detection rates. First, we discuss the effects of our manipulations on inattentional blindness rates, and relevant theoretical and applied implications. Second, we explore applied and methodological issues arising from data gathered using multiple methods for measuring inattentional blindness.

### Testing Boundary Conditions for Inattentional Blindness

Consistent with the predicted role of task demands and observer attentional set, inattentional blindness rates were higher for the task-*irrelevant* unexpected stimulus than the task-*relevant* unexpected stimulus. Across comparisons, the associations between task-relevance and inattentional blindness rates were generally small-to-moderate in size (cf. comparisons based on lenient coding of participants' verbalizations). These findings extend those from basic perception and discrimination research (e.g., [23], [24], [38]) to show that top-down processing contributes to inattentional blindness for complex dynamic stimuli in applied contexts. In the present study, participants' attentional set is likely to have centered on security-related suspicious activity. This attentional set would permit attending to the woman placing a suspicious parcel on the ground (i.e., the *relevant* stimulus), but filter out the pirate (the *irrelevant* stimulus). According to this view, participants failed to detect the pirate because they did not expect to see her.

Alternatively, dissimilarities in the movements (cf. task-relevance) of the two unexpected stimuli may have contributed to the observed difference in inattentional blindness rates. The (task-irrelevant) pirate entered the scene, stood still, and then exited the scene. In contrast the (task-relevant) woman with the parcel entered the scene, bent down to place the parcel on the ground, stood up, and then stood still before exiting the scene. However, it is unlikely that this movement alone would account for such a notable effect. Simons and Chabris [15] found similar detection rates when their unexpected target (gorilla) stopped mid-way through the scene and thumped his chest (50%) and when the gorilla simply walked through the scene (42%). Thus, differences in inattentional blindness are not accounted for solely by minor (or even major) variations in target movement. Further, the same actor served as the task-relevant and -irrelevant stimuli and, thus, these stimuli were matched on other perceptual qualities (e.g., color, size, body shape, general features). This strongly suggests the contribution of observer expectation and task demands (cf. properties of the stimulus itself) to the inattentional blindness rates observed. Importantly, however, even task-relevant targets were vulnerable to inattentional blindness effects. Further, and extending previous work by Drew et al. [37], this pattern held for experienced operators.

We found no evidence that prior experience with the monitoring task inoculated operators against inattentional blindness. In previous research showing that operator experience can reduce susceptibility to inattentional blindness, experience has referred to either familiarity with executing the task being monitored (e.g., playing basketball), or training in the primary monitoring task with stimuli that are highly similar or identical to those used in the critical trial, prior to being exposed to the critical trial. In contrast, experienced operators in the current study were recruited on the basis of their *general experience* in CCTV monitoring (cf. [37], [38]). While they were trained in, and had prior occupational experience of, monitoring security footage, they were not familiar with the specific stimulus materials used in this experiment. On this note, Howard et al. [43] had trained and naïve operators rate CCTV footage for perceived suspiciousness, and found greater uniformity of ratings among trained (cf. naïve) operators. The researchers suggested that this increased uniformity may indicate that training teaches operators “what to look for”. Thus, experience monitoring CCTV footage *in military contexts* may teach operators what to look for *in military contexts*, but any associated benefits for detection performance may not extend to other monitoring contexts. Thus, similar to benefits related to expertise, the benefits of task experience may be highly specified with regard to context.

Demonstrating that general experience with monitoring tasks did not attenuate inattentional blindness has both theoretical and applied implications. From a theoretical standpoint, it prompts consideration of potential mechanisms underlying inattentional blindness. If inattentional blindness is a function of individuals' finite cognitive resources (i.e., the attentional demands of the primary task prevent the detection of the unexpected target), general familiarity with the task either does not (a) reduce the attentional demands of the primary task or (b) alter the allocation of attention (i.e., prevent operators from devoting all of their available attention to the primary task in order to attend to other stimuli in the environment) to the extent required to ameliorate inattentional blindness with complex, dynamic stimuli. Braun [38] found that task experience can attenuate the effects of manipulations designed to impair basic processes underlying detection. Although task experience may facilitate a link between preattentive processing and perceptual report in a basic detection task – improving detection without reducing attentional load – this finding did not generalize to, or this mechanism was not strong enough to ameliorate detection deficits for, this more complex task. Although Drew et al.'s [37] findings also suggested improved detection for experienced operators, their overall pattern of findings prompted them to note that “… expertise does not immunize against inherent limitations of human attention and perception”.

From an applied perspective, demonstrating that experience monitoring security footage did not protect operators against this basic cognitive deficit highlights the robustness of the effect and its relevance for applied security contexts (see also [37]). Further, it highlights the importance of investigating the influence of human factors on effective security monitoring: Simply having an operator (experienced or not) monitor a collection of CCTV screens does not guarantee effective threat detection or enhanced public safety.

Contrary to expectations, task length did not affect inattentional blindness. Previous research has only investigated inattentional blindness during sequences which require attention over relatively short time periods (approx. one minute). However, in a variety of other detection tasks, operators' detection rates have been shown to drop over time (e.g., [8], [29]). Thus, the absence of any effect in the present study is surprising. However, the footage added prior to the critical event (to create the longer clips) was monotonous, and may have permitted effective performance with minimal vigilance. Participants viewing the longer versions of the footage may have reduced their vigilance over time, but re-focused their attention at the start of the critical event. Although this may not be an optimal manipulation of sustained attention, it approximates operationally-relevant monitoring conditions [31].

Alternatively, inattentional blindness may be independent of the length of time for which an individual has been attentive, and simply reflect the direction of attention towards another object, task or event at the time the unexpected stimulus appears. Increasing (decreasing) the *total amount* of attentional resources available when the unexpected stimulus appears will not reduce (increase) inattentional blindness if *all* of these resources remain directed toward the primary task [27]. The present findings extend previous research on inattentional blindness by suggesting that task length (i.e., the length of time for which attention is required) *per se* does not affect inattentional blindness. Consistent with previous work (e.g., [15]) our findings suggest that, even for experienced operators, prolonged monitoring periods are not a necessary precondition for substantial inattentional blindness effects.

### Verbalization vs. Recall Based Measures of Inattentional Blindness, and Criteria for Detection

Inattentional blindness is typically measured using a post-event questionnaire. We compared detection rates based on a typical post-event questionnaire with a novel measure based on participants' real-time verbalizations. While researchers have argued against a memory-based mechanism for inattentional blindness [15], we believed that the method used to assess detection may contribute to detection rates. Specifically, given the established fallibility of recall memory [44], we were concerned that relying on participants' recall of the event (i.e., using post-event questionnaires to measure detection) implicitly introduces a memory confound, and may inflate inattentional blindness rates. The results did not support this position. However, our results did demonstrate that the method used to measure detection affected inattentional blindness rates – albeit in the opposite direction to predictions based on the *inattentional amnesia* hypothesis [40]. Specifically, inattentional blindness rates based on the post-event questionnaire were lower than those based on participants' real-time verbalizations. Care was taken to ensure that the questionnaires used were not suggestive, and information contained in the questionnaire items was not sufficient to allow participants who had not detected the unexpected stimulus to pass the strict criterion for detection. Thus, we do not believe the increased detection rates merely reflect suggestive questioning. However, questionnaire items may have served as recall cues, facilitating participants' memories for the event, and their reporting of the unexpected stimuli. Alternatively, detection rates based on real-time verbalizations may have been lower than those based on the post-event questionnaire because participants experienced difficulty verbalizing the critical event and the unexpected target simultaneously. However, approximately 40% of participants were able to offer a verbalization corresponding to the appearance of the unexpected target, so difficulties associated with concurrent verbalization do not wholly account for the effect. The precise mechanism for this effect is unclear. However, by using a novel, non-memory based index of detection, our results clearly demonstrate that inattentional blindness is a perceptual or attentional deficit, not a product of memory failure [15].

Further to the differences in detection rates based on verbalization and post-event recall data, our data suggested that a number of participants detected the unexpected stimulus but failed to correctly describe/identify it (in both their verbalizations and their post-event recall responses). We used high quality, color clips so these errors are unlikely to reflect the quality of visual information available. This finding has two additional implications. First, although the present research is concerned with inattentional blindness (i.e., detection failures), identification failures are also relevant for security monitoring settings (the ability to not only detect a stimulus, but to identify it and describe it is of obvious practical importance). Instances of detection without identification based on post-event measures may, at least partially, reflect the nature of the questioning. Questions such as “While you were watching the footage, did you notice anything unusual?” may have encouraged participants to confabulate a positive response. Such an effect would be trivial. However, this is not true for the real-time verbalization measure. Teasing apart detection and identification failures, and understanding the conditions contributing to these two types of error will be important in improving monitoring performance.

Second, from a purely methodological perspective, our results demonstrate that when studying inattentional blindness the choice of measurement method and criterion imposed for detection are important. The precise mechanisms underlying inattentional blindness are debatable (see [45]) and, as demonstrated by the present results, failures to report unexpected events may be interpreted as indicating a form of blindness (failure to perceive or detect) or a form of agnosia (a failure to recognize or identify, see [46]) depending on the methodology used. That design and measurement choices affected outcomes is not surprising, but it is a reminder that methodological and analytical choices should reflect the theoretical and applied contexts motivating the research. Further, the method of measurement used and the level of specificity required to constitute detection must be clearly operationalized in research of this nature.

From an applied perspective, there are important distinctions between our testing environment and a typical CCTV control room. In applied settings, observers are often required to monitor several screens simultaneously. Moreover, surveillance systems often produce poor quality images which in turn contribute to difficulties detecting and identifying stimuli [8]. Our participants monitored a single screen with a high quality image. Nonetheless, most participants – even those with experience monitoring security footage – demonstrated substantial inattentional blindness. Demonstrating robust effects under near-optimal conditions, with both naïve and experienced operators, highlights the relevance of the issue for applied settings (where image quality is poorer and multiple screens require attention).

In the current social-political climate, effective security surveillance is essential for public safety, and the protection of critical infrastructure. However, there has been little academic interest in CCTV monitoring despite its obvious role in security settings [9], [47]. A scientific approach to security surveillance research is required, and such an approach must consider human factors in addition to technical capacity. Knowledge gained from such research may influence the design of monitoring systems, and the training given to operators [48]. The present study contributes to this approach by empirically investigating inattentional blindness in the security monitoring context, demonstrating the robustness of the phenomenon, and highlighting important theoretical and practical issue for further investigation.
